# Physicians’ utilization of microbiologic reports and determinants of their preference to order culture in Tikur Anbessa Specialized Hospital, Addis Ababa, Ethiopia

**DOI:** 10.1186/s13104-018-3782-y

**Published:** 2018-09-21

**Authors:** Getachew Alemkere, Getu Gilagil, Teklu Gebrehiwot, Zelalem Tilahun, Hylemariam Mihiretie Mengist

**Affiliations:** 1 Department of Clinical Pharmacy, College of Health Sciences, Addis Ababa University, Addis Ababa, Ethiopia; 2 Department of Pharmacology and Clinical Pharmacy, School of Pharmacy, College of Health Sciences, Addis Ababa University, Addis Ababa, Ethiopia; 3 Department of Clinical Pharmacy, Ayder Referral Hospital, Mekele, Ethiopia; 4 Department of Medical Laboratory Sciences, College of Health Sciences, Debre Markos University, Debre Markos, Ethiopia; 5 Laboratory of Structural Immunology, School of Life Sciences, University of Science and Technology of China (USTC), Hefei, Anhui China

**Keywords:** Physicians’ utilization, Microbiologic reports, TASH

## Abstract

**Objective:**

The main aim of the study was to assess physicians’ utilization of microbiologic reports and determinants of their preference in ordering microbiologic culture among patients with systemic bacterial infection at Tikur Anbessa Specialized Hospital.

**Results:**

Of the total 369 patients observed, 91 (24.7%) had microbiologic reports (culture and gram stain). About 12% of the patients had culture reports of which majority (77.8%) were available after 72 h of the initial antibiotic start. Antimicrobial susceptibility test was done for 83.3% of the positive cultures. Although 99.5% of the patients were initially placed on empiric therapy, adjustment was done in 114 (30.9%) of the patients. Among these patients with adjusted therapy, changes were unrelated to microbiologic reasons in 103 (90.4%) patients. None of these changes were for the reason of streamlining therapy. Prolonged hospital stay (AOR = 2.9, 95% CI 1.2–6.7), senior physician consultation (AOR = 4.1, 95% CI 1.1–17.7) and suspicion of new site of infection (AOR = 2.6, 95% CI 1.1–6.2) were positive independent predictors for physicians’ preference in ordering culture.

**Electronic supplementary material:**

The online version of this article (10.1186/s13104-018-3782-y) contains supplementary material, which is available to authorized users.

## Introduction

Studies indicated that targeting or adjustment of antibiotic treatment according to the results of antimicrobial susceptibility test results lead to decreased antibiotic use, cost and increased therapeutic efficacy [1, 2]. Though microbiologic reports were expected before the initiation of any antibiotic treatment, published microbiologic guidelines do not specifically state when cultures should be drawn for most clinical conditions; thus, it is generally accepted and taken as the quality of care indicator if drawn and reported before antibiotic startup [3–5]. For initial treatment, however, to avoid bad consequences of delayed therapy while waiting microbiologic reports, immediate and adequate/appropriate empiric therapy is generally recommended in the treatment guidelines. Further, these guidelines state that the empiric therapies should be streamlined on the bases of microbiologic cultures [6, 7].

Because of the challenges of determining true bacteremia for positive microbiologic cultures, physicians are highly ignorant to order cultures or tend to order irregularly. On the other hand, because of the high mortality associated with bacteremia, the dangers of undertreating some infections, or concern about using inappropriate antibiotics, physicians tend to order blood cultures liberally [8–10].

Despite this controversy, physicians had better persistently help to improve and rely on the microbiologic investigations in the way that enable to challenge the risks posed by antimicrobial resistance [1–5]. Although the issue of antimicrobial resistance is critically increasing, there is paucity of published data in the study setting. The main aim of this study is; therefore, to assess physicians’ practice in using microbiologic reports and determinants of their preference in ordering microbiologic cultures in TASH. The results of this study may have paramount importance for physicians, policy makers and patients at large in fighting antimicrobial resistance and routine use of microbiologic reports before prescription of antimicrobial agents.

## Main text

### Materials and methods

#### Study setting and context

Institution based prospective observational study design was employed. The study was conducted in TASH from 9 April to 7 July 2014. TASH is a teaching hospital with multiunit services.

#### Population

All patients (with any age range) attending the medical ward including the medical intensive care unit (ICU) of TASH during the study period and who had suspected systemic bacterial (non-mycobacterial) infection were included. Patients with suspected systemic bacterial infections and dispensed with systemic antibacterial drugs in medical ward during the study period were included. Patients taking anti-mycobacterial agents, non-systemic antibacterial agents, and prophylactic antibacterial agents were excluded.

#### Data collection

Data abstraction format adopted from the different literatures was used for data collection. Pilot study was conducted prior to actual data collection and adjustments were done accordingly. Data was abstracted from the patient documentation sheet (patient card) and the attached microbiologic reports. For some ambiguities, however, the bedside nurse and the attending physician had been consulted as necessary. The data collectors were four clinical pharmacy staffs at the hospital and they were trained for 2 days. They were assigned for the collection of data under strict supervision of the principal investigators.

#### Data processing and analysis

The collected data was checked and cleaned for any deficit prior to data entry. Errors in data entry were also checked for accuracy using double data entry technique. Epi info version 7 software was used for data entry and SPSS for windows version 21.0 was used for data analysis. Descriptive statistics were used to summarize data. Binary logistic regression models were used to measure the association of dependent and independent variables.

#### Operational definitions

The following terms are operationally defined for this particular study.

##### Prolonged stay

Patients stayed in the hospital above the median (> 14 days).

##### Antibiotics

Refers to drugs used for systemic bacterial infection.

##### Definitive therapy

The definitive therapy was labeled based on availability of antimicrobial susceptibility test results irrespective of the true or false positivity of microbiologic results.

##### Adjustment

Changes made on the antibiotic/regimen after 48–72 h of the initial therapy that refers to either of following.

- Discontinued: To mean any discontinuation of all antibiotics found to be unnecessary (e.g. no suspected infection).
- Modified: To mean either de-escalation (narrowing by either discontinuation of either agent or using the narrower spectrum option) or broadening (addition or using a much broader spectrum instead or starting a new cycle of treatment after a day and before 7 days of completion of the first course of treatment period) of therapy.

### Results

#### Admission characteristics

**Fig. 1:**
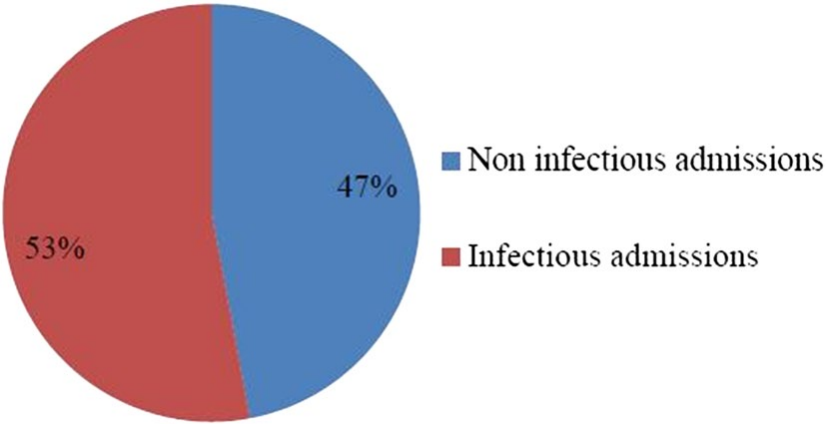
Admission characteristics of hospitalized patients in the internal medicine ward of Tikur Anbessa Specialized Hospital

Six hundred ninety-seven patients (626 in the wards and 71 in the ICU) were admitted to the 120 bed medical ward and 6 bed medical ICU during the data collection period. Among these, 369 patients had suspected systemic bacterial infections during or after admission (Fig. 1).

#### Socio-demographic and disease related factors

Majority of the patients with suspected infection were adults in the age range of 18–64 with mean age of 39.8. The median hospital stay length of the patients was 14 days ranging from 3 to 60 days. More than half of them were females. Above 70% of the patients had suspected infection on admission (Table 1). Pneumonia, 48.0%, was the major infection suspected followed by sepsis, 13.0%, and urinary tract infections, 12.5% (Additional file 1).

**Table 1:** Socio-demographic and disease related characteristics of hospitalized patients with systemic bacterial infection in the internal medicine ward of TASH in 2014, Addis Ababa, Ethiopia

Variables	Frequency (N = 369)	Percent (%)
Age groups (years)		
≤ 17	27	7.3
18–64	287	77.8
≥ 65	55	14.9
Average age of patient ± SD (range)	39.8 ± 18.5 (10–85)	
Sex of patient		
Female	191	51.7
Male	178	48.2
In hospital length of stay	Median 14 days (range 3–60 days)	
Setting		
Medical ICU	42	11.4
Internal medicine	327	88.6
Admission diagnosis		
Infectious	269	72.90
Circulatory	123	33.33
Neoplasm	100	27.10
Signs and symptoms of disease^a^	99	26.83
Endocrine and metabolic	40	10.84
Digestive	30	8.13
Genitourinary	25	6.78
Blood related	26	7.05
Respiratory	27	7.32
Other diagnosis^b^	27	7.32

*SD* standard deviation

^a^Based on International Classification of Disease (ICD 10) criteria referrers to the signs and symptoms of the underlying disease (e.g. hemiparesis, secondary to hypertension) that was not classified elsewhere under the primary admission diagnosis but which were the primary reasons for admission

^b^Drug adverse outcomes (9), seizure/epilepsy (4), gynecology (3), arthritis (2), communicable hydrocephalus (1) cholestatic calculi (1), and injury (2)

#### Initial therapy and evidences for change

Almost all of the patients were initially placed on empiric therapy. The initial therapy was adjusted in 114 (30.9%) of the patients. Among these patients with adjusted antibiotic changes, the reason of change was not due to microbiologic reasons in 103 (90.4%) patients. The top reasons for change were suspicion of new site infection (32.5%), followed by clinical deterioration (20.2%), intravenous to oral drug switch (18.4%) and senior physician consultation (9.6%) respectively. Microbiologic report accounts only for 9.6% of the changes and all these changes were attributed from a positive culture results, except one, from a negative culture. Furthermore, none of the changes were for the reason of streamlining therapy (Additional file 2).

#### Microbiological reports and determinants of physicians’ preference

Of the total 369 patients, 91 (24.7%) had microbiologic reports. Seventy-six (83.5%) of them were reported in the wards, and fifteen (16.5%) in ICU. Ten (11.0%) of them had both Gram stain and culture, 46 (50.5%) had only Gram stain and the remaining 35 (38.4%) had only culture (Additional file 3). Most of the cultures, 35 (77.8%), were reported after the antibiotic startup. From 12 positive cultures, antimicrobial susceptibility test was done for 10 (83.3%) of them (Additional file 4).

Prolonged hospital stay (AOR = 2.9, 95% CI 1.2–6.7), senior physician consultation (AOR = 4.1, 95% CI 1.1–17.7) and suspicion of new site infection (AOR = 2.6, 95% CI 1.1–6.2) were positive, and presence of HIV infection (AOR = 0.1, 95% CI 0.02, 0.98) were negative independent determinants for physicians’ preference in ordering culture (Table 2).

**Table 2:** Logistic regression analysis for physicians’ preference in ordering culture among patients with systemic bacterial infections in the internal medicine wards of TASH TASH in 2014, Addis Ababa, Ethiopia

Variable	Culture ordered		COR (95% CI)	AOR (95% CI)
	No	Yes		
Sex of patient				
Female	176 (92.1)	15 (7.9)	1.00	1.00
Male	148 (83.1)	30 (16.9)	2.4 (1.2, 4.6)**	2.0 (.98, 4.2)
Length of stay				
≤ 14	181 (94.8)	10 (5.2)	1.00	1.00
> 14	143 (80.3)	35 (19.7)	4.4 (2.1, 9.3)***	2.9 (1.2, 6.7)**
On admission HIV infection				
Present	274 (86.2)	44 (13.8)	1.00	1.00
Absent	50 (98.0)	1 (2.0)	0.1 (0.02, 0.9)*	0.1 (0.02, 0.98)*
Febrile neutropenia				
Present	289 (89.2)	35 (10.8)	1.00	1.00
Absent	35 (77.8)	10 (22.2)	2.4 (1.1, 5.2)*	1.5 (0.6, 3.5)
Pleural effusion empyema				
Present	320 (88.4)	42 (11.6)	1.00	1.00
Absent	4 (57.1)	3 (42.9)	5.7 (1.2, 26.4)*	4.4 (0.6, 31.1)
Senior consultation				
No	317 (88.5)	41 (11.5)	1.00	1.00
Yes	7 (63.6)	4 (36.4)	4.4 (1.2, 15.8)*	4.1 (1.1. 17.7)*
Clinical deterioration				
Present	311 (89.1)	38 (10.9)	1.00	1.00
Absent	13 (65.0)	7 (35.0)	4.4 (1.7, 11.7)**	2.7 (0.9, 7.8)
Suspicion of new site infection				
Present	296 (90.2)	32 (9.8)	1.00	1.00
Absent	28 (68.3)	13 (31.7)	4.3 (2.0, 9.1)***	2.6 (1.1, 6.2)*

*COR* crude odds ratio, *AOR* adjusted odds ratio

* P ≤ 0.05; ** P ≤ 0.01; *** P ≤ 0.001

### Discussion

Pneumonia was the most common infection in hospitalized patients in this study which is similar with other studies [11, 12]. Unlike these studies, infection was the primary diagnosis for admission followed by circulatory disorders in the present study. The difference might be due to differences in the prevalence of infectious and non-infectious diseases in different countries.

Empiric therapy was initiated for more than 99% of patients in the wards and all patients in the ICU of the present study. This was in complete disagreement with a study conducted in another teaching hospital [13], where empiric therapy was initiated only for 19.4% of the patients. As per studies, antibiotics ordered empirically were found to be less appropriate than those ordered with evidence of culture and susceptibility reports [14].

Failures to obtain microbiologic cultures represent failed opportunities for guiding antimicrobial therapy [15]. On the other hand, each contaminated culture incurs an additional cost (due to additional diagnostic testing, increased length of stay, unnecessary medication use and associated adverse events) [16]. This implies, besides obtaining microbiologic cultures, appropriate changes according to the microbiologic reports are essential.

One of the important issues in this study is that the microbiologic reports were not appropriately used for therapy adjustment. In the current study all the adjustments were solely attributed to the positive cultures, irrespective of their false or true positivity. Different studies that evaluated outcomes for culture positive and culture negative reports evidenced that patients with culture negative infections differ substantially (had lower severity of illness, hospital mortality, and hospital length of stay) from patients with positive microbiologic cultures [17, 18]. Therefore, antibiotics can safely be discontinued for culture negative reports [1, 2, 17].

Another finding in this study is that most microbiologic cultures (77.8%) were performed after antibiotics were administered. This can potentially decrease culture yield as compared to patients who are not receiving antibiotics [19, 20].

Prolonged in hospital stay (AOR = 2.9, 95% CI 1.2–6.7), senior consultation (AOR = 4.1, 95% CI 1.1–17.7) and suspicion of new site infection (AOR = 2.6, 95% CI 1.1–6.2) were positive, and presence of HIV infection (AOR = 0.1, 95% CI 0.02, 0.98) were negative independent predictors for physicians’ preference in ordering culture. This implies that physicians need microbiologic results when patients stay long indication ineffectiveness of prescribed antibiotics and it is usual that physicians must change regimen when new infection is suspected. Presence of HIV infection negatively associated with physicians’ preference in ordering culture in this study. The possible justification may be the fact that HIV patients may show different opportunistic diseases symptoms due to immunosuppression which leads physicians ignorant at one hand and HIV patients are usually prescribed for prophylaxis irrespective of culture results on the other hand.

### Conclusion

Generally, physicians’ microbiologic utilization in optimizing antimicrobial therapy was not only suboptimal but also performed liberally. They mainly rely on their clinical judgment than initiating microbiologic orders unless for patients with prolonged hospital stay, suspicion of late onset of new infections and senior physician consultation. In this era of increasing antimicrobial resistance, wider use of microbiologic cultures should be encouraged to ensure targeted therapy and cost reduction, particularly with severely ill hosts. However, microbiologic cultures should be used appropriately to avoid unintended consequences. Regimen changes should be based on antimicrobial susceptibility tests and further large scale researches are recommended.

## Limitations

This study has limitations: (1) Specific microbiology specimen sources were mot recorded. (2) The current study did not use any measures to assess the correctness of changes and/or the true or false positivity of microbiologic results.

## Additional files

**Additional file 1.** Types of systemic bacterial infections suspected or proven in hospitalized patients in the internal medicine ward of TASH in 2014, Addis Ababa, Ethiopia.

**Additional file 2.** Initial therapy type, type of change and the reasons for change in hospitalized patients in the internal medicine ward of TASH in 2014, Addis Ababa, Ethiopia.

**Additional file 3.** The number and type of microbiologic reports available for infected patients followed in medical ward of TASH in 2014, Addis Ababa, Ethiopia.

**Additional file 4.** Microbiologic reports available and/or used for therapy adjustment in hospitalized patients in the internal medicine ward of TASH in 2014, Addis Ababa, Ethiopia.

## Abbreviations

AOR: adjusted odds ratio
AST: antimicrobial susceptibility test
COR: crude odds ratio
ICU: intensive care unit
TASH: Tikur Anbessa Specialized Hospital
