# Supplementary material for: Physicians’ utilization of microbiologic reports and determinants of their preference to order culture in Tikur Anbessa Specialized Hospital, Addis Ababa, Ethiopia
Source: BMC Res Notes. 2018 Sep 21;11:675. doi: 10.1186/s13104-018-3782-y (PMC6151033; doi:10.1186/s13104-018-3782-y)
Supplement: Supplementary file 1 — Additional file 1. Types of systemic bacterial infections suspected or proven in hospitalized patients in the internal medicine ward of TASH in 2014, Addis Ababa, Ethiopia. [file 13104_2018_3782_MOESM1_ESM.docx]

**Additional file 1: Types of systemic bacterial infections suspected or proven in hospitalized patients in the internal medicine ward of TASH in 2014, Addis Ababa, Ethiopia.**

Pneumonia, 48.0 %, was the major infection suspected followed by sepsis, **13.0**%, and urinary tract infections, 12.5 %.

| **Bacteria diagnosed** | **Frequency** | **Percentage (%)** |
| --- | --- | --- |
| Pneumonia | 155 | 48.0 |
| Urinary Tract Infection | 46 | 12.47 |
| Sepsis | 48 | 13.01 |
| Fever of neutropenia | 45 | 12.20 |
| Meningitis | 23 | 6.23 |
| Abscess | 18 | 4.88 |
| Spontaneous Bacterial Peritonitis | 13 | 3.52 |
| Gastroenteritis | 11 | 2.98 |
| Diabetic foot ulcer | 11 | 2.98 |
| Infective endocarditis | 11 | 2.98 |
| Skin infections | 10 | 2.71 |
| Paranumonic effusion/empyema | 7 | 1.90 |
| *Other Bacterial infections | 45 | 12.20 |

^*Acute bronchitis, Acute Post Streptococcal Glomerulonephritis, Acute febrile illnesses, Chronic diarrhea, Cough, Emphysema, H. pylori, Intra-abdominal infections, lymphadenitis (Pyogenic), Odontogenic infections, Osteomyelitis, Otitis Media, Pneumothorax, Sore throat & acute bronchitis^
