# Supplementary material for: Physicians’ utilization of microbiologic reports and determinants of their preference to order culture in Tikur Anbessa Specialized Hospital, Addis Ababa, Ethiopia
Source: BMC Res Notes. 2018 Sep 21;11:675. doi: 10.1186/s13104-018-3782-y (PMC6151033; doi:10.1186/s13104-018-3782-y)
Supplement: Supplementary file 2 — Additional file 2. Initial therapy type, type of change and the reasons for change in hospitalized patients in the internal medicine ward of TASH in 2014, Addis Ababa, Ethiopia. [file 13104_2018_3782_MOESM2_ESM.docx]

**Additional file 2: Initial therapy type, type of change and the reasons for change in hospitalized patients in the internal medicine ward of TASH in 2014, Addis Ababa, Ethiopia**

Almost all of the patients were initially placed on empiric therapy. The initial therapy was adjusted in 114 (30.9%) of the patients. Among these patients with adjusted antibiotic changes, the reason of change was not due to microbiologic reasons in 103 (90.4%) patients. The top reasons for change were suspicion of new sit of infection (32.5%), followed by clinical deterioration (20.2%), IV to Po switch (18.4%) and senior consultation (9.6%) respectively. Microbiologic report accounts only 9.6 % of the changes and all these changes were attributed from a positive culture results, except one, from a negative culture. Furthermore, none of the changes were for the reason of streamlining therapy.

| **Variables** | **Frequency** | **Percent (%)** |
| --- | --- | --- |
| **Initial therapy type (N=369)** |  |  |
| Empiric | 367 | 99.5 |
| Definitive | 2 | 0.5 |
| Changes to the initial therapy (N=369) |  |  |
| No change | 255 | 69.1 |
| Modified | 102 | 27.6 |
| Discontinued | 12 | 3.2 |
| Reasons of change (n=114) |  |  |
| *Microbiologic report | 11 | 9.6 |
| Suspicion of new Site of infection | 37 | 32.5 |
| Clinical deterioration | 23 | 20.2 |
| IV to PO change | 21 | 18.4 |
| Senior consultation | 11 | 9.6 |
| **Other reasons | 11 | 9.6 |

^*Except one, all were attributed from positive cultures only; **Discontinued after ruling out infection (5) changed for cost reason (4), changed for drug side effect (2)^
