# Supplementary material for: Physicians’ utilization of microbiologic reports and determinants of their preference to order culture in Tikur Anbessa Specialized Hospital, Addis Ababa, Ethiopia
Source: BMC Res Notes. 2018 Sep 21;11:675. doi: 10.1186/s13104-018-3782-y (PMC6151033; doi:10.1186/s13104-018-3782-y)
Supplement: Supplementary file 3 — Additional file 3. The number and type of microbiologic reports available for infected patients followed in medical ward of TASH in 2014, Addis Ababa, Ethiopia. [file 13104_2018_3782_MOESM3_ESM.docx]

**Additional file 3: The number and type of microbiologic reports available for infected patients followed in medical ward of TASH in 2014, Addis Ababa, Ethiopia.**

Of the total 369 patients, 91(24.7%) had microbiologic reports. Seventy-six (83.5%) of them were reported in the wards, and fifteen (16.5%) in ICU. Ten (11.0%) of them had both gram stain and culture, 46 (50.5%) had only gram stain and the remaining 35 (38.4%) had culture only.

| Microbiologic reports | Wards **(N=332)** | ICU **(N=37)** | Total **(N=369)** |
| --- | --- | --- | --- |
| Culture reports only | 30 (9.04) | 5 (13.51) | 35 (9.49) |
| Gram stain reports only | 37 (11.14) | 9 (24.32) | 46 (12.47) |
| Culture & Gram stain reports | 9 (2.71) | 1 (2.70) | 10 (2.71) |
| Total microbiologic reports | 76 (22.89) | 15 (40.54) | 91 (24.66) |
