# Supplementary material for: Physicians’ utilization of microbiologic reports and determinants of their preference to order culture in Tikur Anbessa Specialized Hospital, Addis Ababa, Ethiopia
Source: BMC Res Notes. 2018 Sep 21;11:675. doi: 10.1186/s13104-018-3782-y (PMC6151033; doi:10.1186/s13104-018-3782-y)
Supplement: Supplementary file 4 — Additional file 4. Microbiologic reports available and/or used for therapy adjustment in hospitalized patients in the internal medicine ward of TASH in 2014, Addis Ababa, Ethiopia. [file 13104_2018_3782_MOESM4_ESM.docx]

**Additional file 4: Microbiologic reports available and/or used for therapy adjustment in hospitalized patients in the internal medicine ward of TASH in 2014, Addis Ababa, Ethiopia.**

Most of the cultures, 35 (77.8%), were reported after the antibiotic startup. For 12 positive cultures, antibacterial susceptibility test was done for 10 (83.3%).

| Variables | Total frequency of evidences reported (%) | Microbiologic reports available during new therapy initiation (within 72 h) | | Reports used for therapy adjustment |
| --- | --- | --- | --- | --- |
|  |  | Initial | Adjusted |  |
| Suspicion of infection | 369 (100) *** | n= 369 (100%) | n=36 (31.6%) | - |
| Gram Stain report | 56 (15.2) *** | n= 12 (3.3%) | n= 8 (7.0%) | - |
| Positive stain report | 8 | - | - | - |
| Culture report | 45 (12.2) *** | n= 2 | n= 16 (14.0%) | 11 |
| Positive culture report | 12 (26.7) *** | - | - | 10 |
| Culture-susceptibility report | 10 | 1 | 1 | 10 |

^* NB: The consecutive bottom column values were included in the adjacent top value list^
